# Salicylic Acid Is Required for Broad-Spectrum Disease Resistance in Rice

**DOI:** 10.3390/ijms23031354

**Published:** 2022-01-25

**Authors:** Bingbing Liang, Han Wang, Ce Yang, Luyao Wang, Linlu Qi, Zejian Guo, Xujun Chen

**Affiliations:** Key Laboratory of Pest Monitoring and Green Management, MOA, Joint Laboratory for International Cooperation in Crop Molecular Breeding, Department of Plant Pathology, China Agricultural University, Beijing 100193, China; liangbb2121@163.com (B.L.); hanwang@nwafu.edu.cn (H.W.); chinayangce@163.com (C.Y.); luyaowang0923@163.com (L.W.); qilinlu1987@126.com (L.Q.)

**Keywords:** immunity, dioxygenase, hydroxylation, jasmonic acid, *Oryza sativa*, salicylic acid

## Abstract

Rice plants contain high basal levels of salicylic acid (SA), but some of their functions remain elusive. To elucidate the importance of SA homeostasis in rice immunity, we characterized four rice SA hydroxylase genes (*OsSAHs*) and verified their roles in SA metabolism and disease resistance. Recombinant OsSAH proteins catalyzed SA in vitro, while OsSAH3 protein showed only SA 5-hydroxylase (SA5H) activity, which was remarkably higher than that of other OsSAHs that presented both SA3H and SA5H activities. Amino acid substitutions revealed that three amino acids in the binding pocket affected SAH enzyme activity and/or specificity. Knockout *OsSAH2* and *OsSAH3* (*sah*KO) genes conferred enhanced resistance to both hemibiotrophic and necrotrophic pathogens, whereas overexpression of each *OsSAH* gene increased susceptibility to the pathogens. *sah*KO mutants showed increased SA and jasmonate levels compared to those of the wild type and *OsSAH*-overexpressing plants. Analysis of the *OsSAH3* promoter indicated that its induction was mainly restricted around *Magnaporthe oryzae* infection sites. Taken together, our findings indicate that SA plays a vital role in immune signaling. Moreover, fine-tuning SA homeostasis through suppression of SA metabolism is an effective approach in studying broad-spectrum disease resistance in rice.

## 1. Introduction

Plants have a sophisticated defense system against pathogen invasion. A general defense is the pathogen-associated molecular pattern (PAMP)-triggered immunity (PTI), initiated by the recognition of PAMPs via pattern recognition receptors (PRRs) [1]. Conversely, a more specific defense is the effector-triggered immunity (ETI), activated by host resistance genes that recognize effectors secreted by pathogens to suppress PTI. Phytohormones, such as salicylic acid (2-hydroxy benzoic acid, SA) and jasmonic acid (JA), are important compounds in the immune signaling pathways. SA accumulation is elevated in many plants owing to pathogen infection, and it activates many downstream responsive genes, as well as genes encoding PAMP receptors and co-receptors [2].

SA is synthesized from shikimate through either the isochorismate or phenylpropanoid pathway [2,3]. Chorismate is converted to isochorismate by isochorismate synthase (ICS). *Arabidopsis* genome contains two *ICS* genes, responsible for 90% of SA synthesis under stimulation by pathogen challenge or UV radiation [4]. Isochorismate is conjugated with glutamate to form isochorismate-9-glutamate by PBS3 (avrPphB susceptible3) and is then converted into SA by EPS1 (enhanced susceptibility) or via spontaneous decomposition [5,6]. Phenylalanine, derived from chorismate, can be converted into SA through the phenylpropanoid biosynthesis pathway. Phenylalanine ammonia-lyase (PAL) catalyzes the conversion of Phe to *trans*-cinnamic acid (tCA), which is oxidized to benzoic acid (BA) by abnormal inflorescence meristem1 (AIM1), functioning as a beta-oxidation enzyme to shorten the side chain. BA is then hydroxylated into SA by an unidentified enzyme.

SA can be modified reversibly by conjugation with amino acids (or glucose) and methylation. Uridine diphosphate glycosyltransferases (UGTs), UGT74F1, UGT74F2, and UGT76B1 catalyze SA glycosylation to salicyloyl glucose ester or SA-O-β-D-glucoside (SAG), which is stored in the vacuole [7,8]. Moreover, SA can be modified through irreversible ways. In *Arabidopsis*, the *downy mildew resistant* (*dmr6*) mutant has increased resistance against the oomycete pathogen *Hyaloperonospora parasitica*, and *DMR6* is cloned as a member of 2-oxoglutarate-dependent dioxygenases (2OGDs) [9]. DMR6 and its close homolog DLO1 (DMR6-like oxygenase1) act as partially redundant, but distinct, suppressors of immunity [10]. Later, DMR6 and DLO1/S3H1 were identified as SA 5-hydroxylase (SA5H) and 3-hydroxylase (SA3H), respectively [11,12]. In the *dmr6 dlo1/s3h1* double mutant, the SA level is further elevated compared with that of the *dmr6* single mutant, leading to immunity to pathogens and strong growth retardation [10,12]. The SA hydroxylation products of 2,3- (2,3-DHBA) and 2,5-dihydroxy BA (2,5-DHBA) can be conjugated with glucose by UGT76D1 or with xylose by UGT89A2 [13,14]. Interestingly, overexpression of *UGT76D1* increases SA biosynthesis and disease resistance, suggesting that DHBA glycosylation may function as a positive feedback activation loop of SA [14]. More recently, SA was converted to catechol in the tomato by an SA 1-hydroxylase, which catalyzes oxidative decarboxylation and evolves specifically within the Solanaceae family [15].

Enzymes 2OGDs utilize 2-oxoglutarate (2OG) and molecular oxygen as co-substrates and ferrous iron Fe(II) as a cofactor to oxidize a substrate through an Fe(IV)-oxo intermediate [16]. Fe(II) is chelated with a facial triad formed by H-D/E-H residues located within a double-stranded β-helix fold [16,17]. Meanwhile, the 5-carboxylate terminal of 2OG is bound to RxS in *Arabidopsis* feruloyl-CoA 6’-hydroxylase1 (F6’H1) [18]. The 2OGD enzymes catalyze a wide array of reactions, including desaturation, demethylation, epimerization, hydroxylation, and ring cleavage. The DOXC group of 2OGDs in plants plays an important role in the homeostasis of phytohormones such as auxin, ethylene, gibberellin, JA, and SA [19,20,21]. Dioxygenase for auxin oxidation catalyzes the conversion of indole-3-acetic acid (IAA) into bio-inactive 2-oxoindole-3-acetic acid [22] and jasmonate-induced oxygenase (JOX) converts JA into bio-inactive 12-OH-JA [23].

SA biosynthesis in rice is also estimated through the isochorismate and phenylpropanoid pathways, in which OsPALs catalyzing the first step of the phenylpropanoid route have been characterized to be important for disease resistance [3]. In the rice *aim1* mutant, accumulation of SA and its associated reactive oxygen species (ROS) in roots is reduced, leading to retardation of root growth [24]. Double knockdown of *OsWRKY62* and *OsWRKY76* (dsOW62/76), two transcriptional repressor genes, increases the SA and JA levels and broad-spectrum disease resistance, and partially compromises the rice *aim1* phenotype [24,25]. Rice contains high basal levels of SA compared to those of *Arabidopsis* and tobacco [26]. Interestingly, decreasing endogenous SA by expressing the bacterial salicylate hydroxylase *NahG* gene revealed that SA plays an important role in protecting rice plants from oxidative stress [26]. However, the biological importance of endogenous SA in rice remains elusive. In this study, we characterized four rice SA hydroxylase genes (*OsSAHs*) and showed that endogenous SA accumulation is crucial for rice disease resistance.

## 2. Results

### 2.1. OsSAH Proteins Have SA 3- and/or 5-Hydroxylase Activity In Vitro

**Figure 1:**
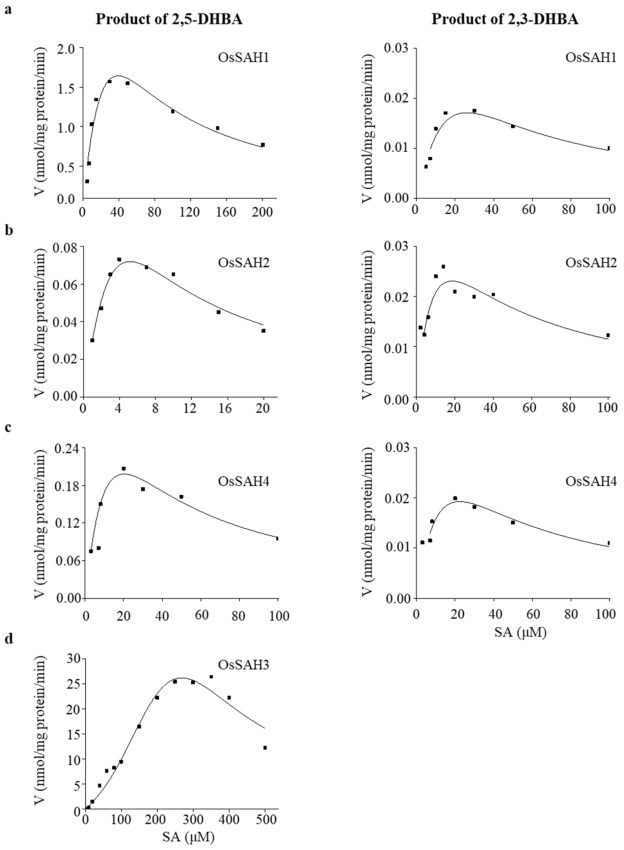
Conversion of SA to 2,3- and/or 2,5-DHBA by recombinant OsSAHs. Kinetics of recombinant OsSAH1 (**a**), OsSAH2 (**b**), OsSAH4 (**c**), and OsSAH3 (**d**) proteins detected by LC–MS/MS using SA as the substrate. Kinetic curves were obtained from the reactions at pH 6.8 and 40 °C for 30 min.

OsSAH recombinant proteins purified from *Escherichia coli* were tested for enzymatic activity using SA, BA, tCA, or caffeic acid as substrates. Hydroxylase activity was only detectable on the SA substrate analyzed using LC–MS/MS, suggesting high substrate specificity of OsSAH proteins. OsSAH1–4 proteins had both SA 3- (SA3H) and 5-hydroxylase (SA5H) activity except of OsSAH3, which converted SA to 2,5-DHBA only. The biochemical parameters of OsSAH1–4 proteins are listed in Table 1. Each OsSAH protein showed higher *Kcat* and *Kcat*/*Km* values for SA5H than for SA3H activity, suggesting that the enzyme favored the conversion of SA to 2,5-DHBA in vitro. In addition, OsSAH proteins exhibited substrate SA inhibition, showing a parabolic velocity curve (Figure 1). OsSAH3 was used to determine the optimal enzymatic conditions owing to its large *Km* value, and it showed an optimal pH of approximately 6.8 and a temperature of 40 °C under the assay conditions (Figure S1).

### 2.2. Key Amino Acids of SAHs Affect Enzymatic Activity

*OsSAH2* and *OsSAH3*, localized tail-to-tail in chromosome 4, are probably gained by duplication; however, the two resulting proteins showed different enzymatic activity and specificity (Table 1). To gain information on substrate specificity, we used 3- and 5-position-occupied SA derivatives and 2-Cl-BA as substrates to check the products formed by OsSAH2 and OsSAH3 enzymes. OsSAH2 catalyzed both 3-Cl-SA and 5-Cl-SA to produce the corresponding 3-Cl-5-OH-SA and 3-OH-5-Cl-SA although in very low amounts, whereas OsSAH3 only showed a 5-hydroxylation product (Figure S2). When 2,3-DHBA, 2,5-DHBA, and 2-Cl-BA were used separately as substrates, hydroxylation products were not detected for OsSAH2 or OsSAH3 proteins. These results suggest that OsSAH2 and OsSAH3 bind restrictively with substrates for product specificity and that nucleophilic substitution on the benzene moiety favors hydroxylation.

**Figure 2:**
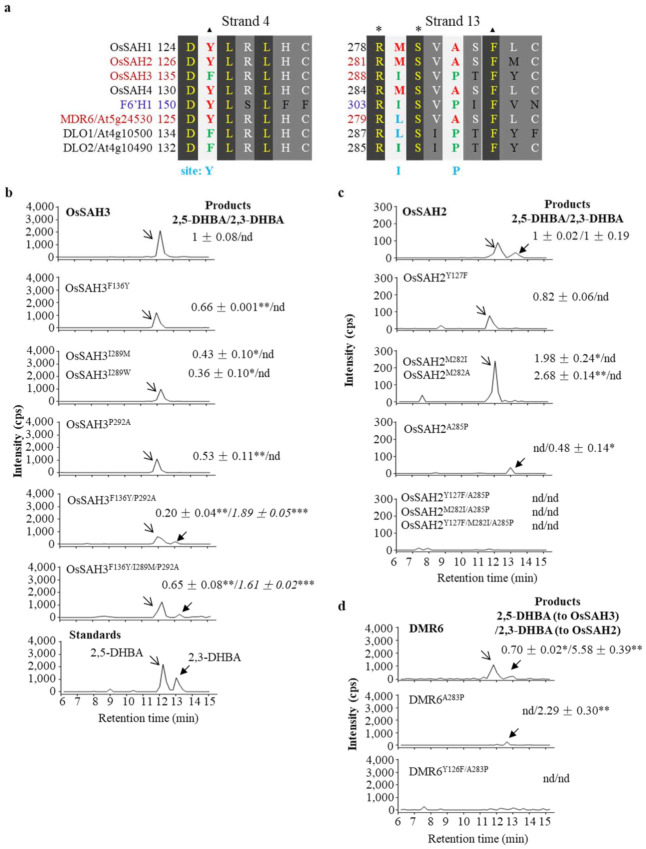
Analysis of amino acids affecting SAH activity. (**a**) Diagrams of β-sheet 4 and 13. ▲ sites potencial binding with SA. * sites association with 2-oxoglutarate. Sequences of rice and *Arabidopsis* SAHs were aligned with F6’H1. Site Y, I, and P were the positions for mutation analysis. The number indicating the position of the first amino acid aligned. Relative enzyme activities of OsSAH3 (**b**), OsSAH2 (**c**), DMR6 (**d**), and their mutants were shown. The activity of the wild type protein was set as 1 and the mutant activity was compared with its own wild type, unless otherwise indicated. The 2,3-DHBA produced by OsSAH3 mutants ((**b**), the number in italic) was in related to that produced by OsSAH2 (**c**). In the case of DMR6 (**d**), the 2,5-DHBA activity was compared with OsSAH3, and the 2,3-DHBA activity was compared with OsSAH2. Values are given as means ± SD of three biological replicates. Asterisks indicate statistically significant differences compared with the wild type protein (Student’s *t*-test; *, *p* < 0.05; **, *p* < 0.01; ***, *p* < 0.001). nd for not detection.

Sequence alignments of rice SAHs with F6’H1 revealed amino acid differences around the substrate-binding sites a and Figure S3). The conserved RxS site is associated with 2OG, whereas the Phe residue (three amino acids next to RxS motif) and the upstream Tyr (designated as Y site for simplicity) in F6’H1 interacts respectively with the ferrule ring via π stacking and the thiocarboxylate group of feruloyl-CoA [18]. We estimated the amino acids near the substrate-binding sites that may affect the SAH enzyme activity and specificity. Three amino acids were exchanged between OsSAH2 and OsSAH3 at the corresponding positions. Each mutation in OsSAH3 (OsSAH3^F136Y^, OsSAH3^I289M^, and OsSAH3^P292A^) significantly suppressed the conversion of SA to 2,5-DHBA compared with the wild type OsSAH3 (Figure 2b). The production of 2,5-DHBA was further reduced in the double mutant OsSAH3^F136Y/P292A^ (Figure 2b); however, 2,3-DHBA was produced at the level similar to that of OsSAH2, suggesting that the two substituted amino acids favor 2,3-DHBA production. Exchanges of all three amino acids (OsSAH3^F136Y/I289M/P292A^) significantly elevated 2,5-DHBA production compared with OsSAH3^F136Y/P292A^ and maintained 2,3-DHBA formation (Figure 2b).

In contrast to the mutation at site I of OsSAH3 that lowers 2,5-DHBA production, we noticed that OsSAH2^M282I^ yields a higher amount of 2,5-DHBA than that of OsSAH2 and completely inhibited 2,3-DHBA production, suggesting that the amino acid with a short side chain at site I of OsSAH2 prefers 2,5-DHBA formation, which was confirmed by the mutation of Met to Ala (OsSAH2^M282A^). Interestingly, OsSAH2^Y127F^ mutant showed a similar level of SA5H activity as that of OsSAH2 but without SA3H activity (Figure 2c). In contrast, OsSAH2^A285P^ mutant produced only a low amount of 2,3-DHBA product compared with the wild type protein, whereas further amino acid substitutions on the OsSAH2^A285P^ background did not lead to detectable SA3H and SA5H activity (Figure 2c), implying that the A^285^ residue in OsSAH2 is a key amino acid for the enzymatic activity and specificity.

*Arabidopsis* DMR6 protein is more similar to *OsSAH2* than to *OsSAH3,* but shows only SA5H activity, as determined by the HPLC method [12] (Figure 2a). Interestingly, SA5H activity was completely absent in the DMR6^A283P^ mutant, similar to the corresponding OsSAH2 mutant, and SA3H activity was markedly suppressed, as determined by the LC–MS/MS method (Figure 2d). The DMR6^Y126F/A283P^ double mutant produced undetectable products (Figure 2d).

Structural simulation showed that OsSAH3 gives only docking conformation for 2,5-DHBA production (Figure S4a). The OsSAH3^F136Y^ mutation changed the orientation of the R^138^ side chain and hindered its hydrogen bond with 2-OH of SA, increasing the distance between iron and the C5 position of SA (Fe–C5, for simplicity) and SA docking energies, whereas OsSAH3^P292A^ substitution led to SA position shift and slightly increased the Fe–C5 distance (Figure S4a,b). The OsSAH3^F136Y/P292A^ double mutant provided SA docking in two conformations, but with higher energies and longer distances between iron and the potential hydroxylation positions than those of OsSAH3 (Figure S4a,b), which is consistent with the production of 2,3- and 2,5-DHBAs (Figure 2b). Both OsSAH2 and DMR6 bound with SA in two conformations in which the 2,5-DHBA product is preferred, as estimated from the docking energies and Fe–C5 distances (Figure S4d,e). The higher activity of DMR6 than that of OsSAH2 was also supported by these parameters.

### 2.3. Patterns of OsSAH Expression and Phenolic Accumulation

**Figure 3:**
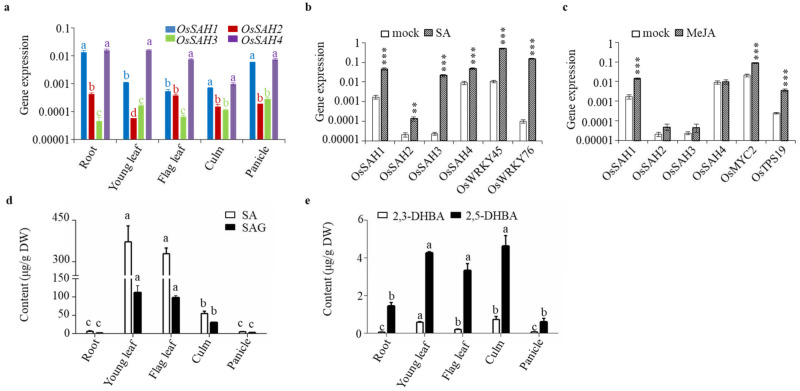
Expression of *OsSAHs* and accumulation of SA and its related compounds. *OsSAH1–4* expression in different tissues (**a**), induction by SA (**b**), and MeJA (**c**). Roots and young leaves were from ten-day-old seedlings cultured in 1/2 MS liquid medium. Flag leaves, culms, and panicles were from plants of three weeks post heading grown in the paddy field. Ten-day-old seedlings cultured hydroponically were treated with 500 μM SA or 100 μM MeJA in 5 mM MES buffer for 6 h. For the mock treatment, the seedlings were received the same volume of DMSO solvent. Gene expression was determined by qRT-PCR using *OsUBQ* as the reference gene. (**d**) SA and SAG, (**e**) 2,3- and 2,5-DHBA. Values are means ± SD (*n* = 3). Asterisks indicate statistically significant differences compared with the corresponding mock using student’s *t*-test (**, *p* < 0.01; ***, *p* < 0.001). Columns marked with different letters (a–c) indicate significant differences among the same tissue (**a**), and the same compound (**d**), as analyzed by the SPSS software (Duncan’s multiple range test, α = 0.05).

*OsSAH* gene expression was observed in different rice tissues (Figure 3a). *OsSAH1* and *OsSAH4* expression levels were generally higher than those of OsSAH2 and OsSAH3 in the same tissues tested, and the transcriptional level of *OsSAH3* in the root was the lowest. As the substrate of OsSAHs, applied SA strongly induced the expression of *OsSAH1–4* and SA-inducible marker genes, i.e., *OsWRKY45* and *OsWRKY76* (Figure 3b). A similar induction of these genes was observed following treatment with benzothiadiazole S-methyl ester (BTH), an SA functional analog (Figure S5). Interestingly, the expression of *OsSAH1* was also markedly induced by methyl jasmonate (MeJA) along with the JA-responsive genes *OsMYC2* and *OsTPS19* (Figure 3c) [27], implying that JA potentially participates in the regulation of SA homeostasis.

Determination of phenolics showed that free SA content was the highest in young leaves among the tissues tested, and the accumulation pattern of SAG was quite similar to that of SA in the same tissues (Figure 3d). The content of free 2,5-DHBA was much higher than that of 2,3-DHBA in the same tissues examined (Figure 3e). 

### 2.4. Knockout OsSAH2–3 Genes Enhance Resistance against Hemibiotrophic and Necrotrophic Pathogens

**Figure 4:**
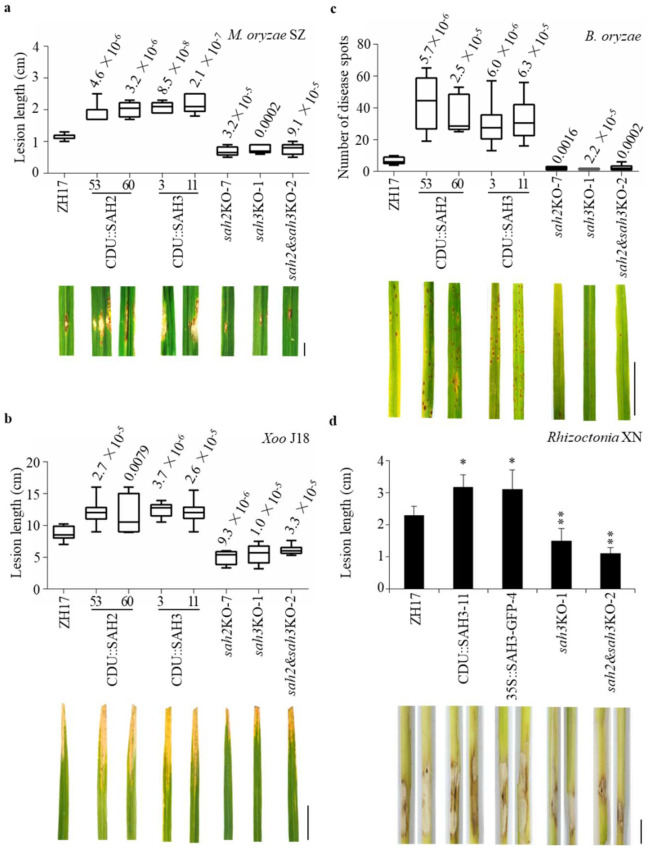
Knockout of *OsSAH2–3* genes conferred resistance to both hemibiotrophic and necrotrophic pathogens. Disease symptoms and lesion lengths or lesion numbers of the transgenic and wild type ZH17 plants were shown. (**a**) Disease phenotypes and lesion lengths of *M. oryzae*. Plants of the tillering stage (about three-month-old) were injected with hemibiotrophic fungus *M. oryzae* SZ spores (1 × 10^5^ conidia/mL). The disease segments were photographed and quantified at 9 d after the injection. (**b**) Disease phenotypes and lesion lengths of *Xoo*. Three-month-old rice plants were inoculated with a hemibiotrophic bacterium *Xoo* J18 strain. Disease symptoms were photographed and evaluated at 18 d after the challenge. (**c**) Disease phenotypes and severity of *B. oryzae*. The transgenic and ZH17 control plants were grown in the paddy field of the experimental station and infected naturally. Evaluation of disease severity and photography taken were conducted about four months after the seed germination in year 2019. Disease numbers were determined on 20 cm of each leaf started 2 cm from the tip (*n* = 10). (**d**) Disease phenotypes and lesion lengths of *Rhizoctonia solani*. Five-week-old rice plants were inoculated with necrotrophic fungus *Rhizoctonia* XN isolate. A slice of filter paper containing the *Rhizoctonia* mycelia was pinned around the sheath bottom. Lesion lengths were measured 4 d after the inoculation. The median was the cross line in each boxplot showing the lesion length (**a**,**b**), and lesion number (**c**) distributions. Experiments were biologically repeated twice with similar results *p* value evaluated using the student’s *t*-test is above the boxplot. Values (**d**) are means ± SD (*n* = 5). Significance was evaluated using the student’s *t*-test (*, *p* < 0.05; **, *p* < 0.01). Prefix CDU for overexpressing gene; suffix KO for knockout gene; and ZH17 for wild type plant. Bar = 1 cm (**a**,**d**), 5 cm (**b**,**c**).

*OsSAH2* and *OsSAH3* were selected for generation of overexpressing and knocking out plants (Figure S6 for gene knockout information). The transgenic and control plants were inoculated with the hemibiotrophic *Magnaporthe oryzae* SZ, a virulent rice blast fungus strain, by foliar spraying of spores on three-week-old plants. Overexpressing *OsSAH2–3* (CDU::SAH2–3, controlled by a *ubiquitin* promoter) plants were more susceptible to the SZ strain than ZH17 control plants, whereas the *OsSAH*-knockout mutants showed enhanced resistance to the pathogen (Figure S7). We also tested blast resistance at the rice tillering stage by injecting SZ spores into rice sheaths. Plants overexpressing each *OsSAH* gene showed increased lesion length compared to those of ZH17 control (Figure 4a). In contrast, enhanced resistance against the rice blast pathogen was observed in the mutants with single and double knockout *OsSAH* (*sah*KO) genes. Furthermore, we examined whether *OsSAH* genes were involved in resistance to the hemibiotrophic *Xanthomonas oryzae* pv. *oryzae* (*Xoo*) J18 strain, which causes the bacterial leaf blight. The lesion lengths were significantly shorter in the *sah*KO plants and longer in the overexpressing lines than in the ZH17 control (Figure 4b), suggesting that the suppression of SA metabolism also increases resistance to the bacterial pathogen.

It is often reported that SA promotes resistance against biotrophic and hemibiotrophic pathogens, and that JA is a key player in facilitating the activation of plant defense against necrotrophic pathogens [28]. *Bipolaris oryzae* is a necrotrophic fungal pathogen that causes the rice brown spot. We observed that *OsSAH*-overexpressing plants exhibited more severe disease symptoms than ZH17 control plants at the tillering stage under natural infection conditions; moreover, the knockout mutants of *OsSAH2–3* showed elevated resistance against *B. oryzae* (Figure 4c and Figure S8a). Afterwards, the transgenic and control plants were inoculated with the spores of *B. oryzae* LW isolate. The responses of the *OsSAH2–3* transgenic plants were similar to those caused by a natural infection of *B. oryzae*, revealing that *OsSAHs* negatively regulated disease resistance against the brown spot pathogen (Figure S8b).

To further assess the effects of SA homeostasis on necrotrophic pathogens, we inoculated some of the transgenic plants with *Rhizoctonia solani*, a soil-borne pathogen with a necrotrophic lifestyle that causes the sheath blight disease. Lesion lengths caused by the challenge of *R. solani* XN strain were markedly reduced upon knockout of *OsSAH3* (*sah3*KO), and both *OsSAH2* and *OsSAH3* (*sah2*&*sah3*KO) plants, whereas the lesion lengths were increased in the CDU::SAH3- and 35S::SAH3-GFP-overexpressing plants (Figure 4d). Furthermore, the plants harboring *OsSAH3*-overexpressing constructs were infected with a weak virulent uninucleate *Rhizoctonia* JN isolate [29]. *OsSAH3*-overexpressing plants were more susceptible to JN isolate than the ZH17 control plants (Figure S9). These results collectively indicate that decreased SA levels elevate rice susceptibility to both hemibiotrophic and necrotrophic pathogens.

### 2.5. Changes of OsSAHs Expression Alter SA Homeostasis

**Figure 5:**
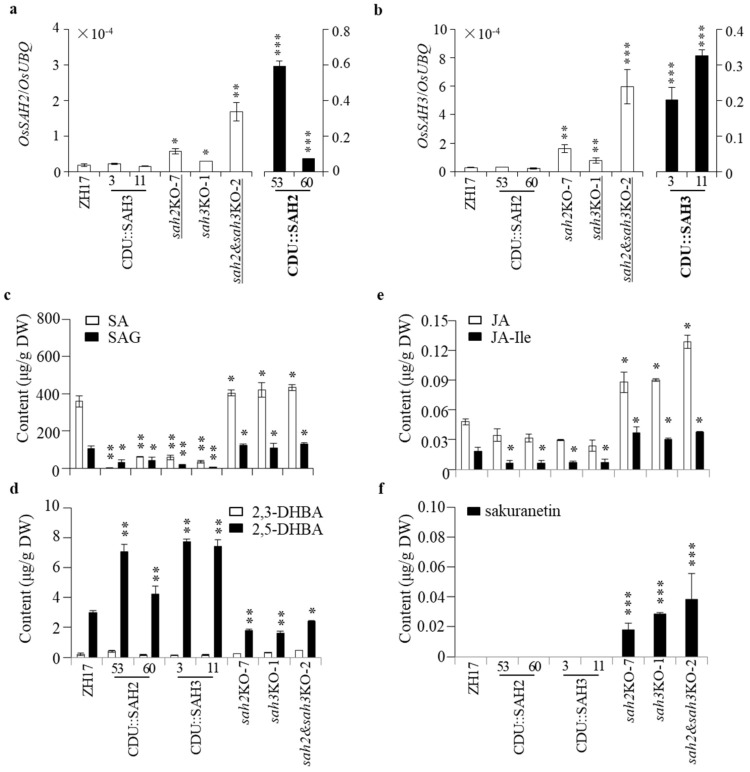
Change of *OsSAH* expression affects SA homeostasis. Expressions of *OsSAH2* (**a**) and *OsSAH3* (**b**) in the transgenic and ZH17 plants. The leaves of three-week-old rice plants were sampled for total RNA and compound isolation. Gene expression was determined by qRT-PCR using *OsUBQ* as the reference gene. Accumulation of SA and SAG (**c**), 2,3- and 2,5-DHBA (**d**), JA and JA-Ile (**e**), sakuranetin (**f**). The amounts of compounds were determined by LC–MS/MS using D_5_BA as an internal standard. Values are given as means ± SD (*n* = 3). Asterisks indicate statistically significant differences compared with ZH17 using the student’s *t*-test (*, *p* < 0.05; **, *p* < 0.01; ***, *p* < 0.001). Prefix CDU for overexpressing gene; suffix KO for knockout gene; and ZH17 for wild type plant. Words underlined, detection of the mutant transcript; words in bold, detection including the exogenous transcript.

Expression of *OsSAH2* and *OsSAH3* was increased in the double knockout (*sah2*&*sah3*KO) and the single-knockout mutants, and their expressions were dramatically elevated in its own overexpressing lines (Figure 5a,b). The content of SA, as the substrate of OsSAHs, was remarkably reduced in *OsSAH*-overexpressing plants and increased in *sah*KO mutants (Figure 5c). The accumulation of 2,5-DHBA product was increased in *OsSAH*-overexpressing lines, whereas the content change of 2,3-DHBA was minor (Figure 5d). The increase in SA accumulation and *OsSAHs* expression in the *sah*KO mutants revealed that a new SA homeostasis was reached.

JA and its bioactive jasmonoyl-L-isoleucine (JA-Ile) play important roles in rice disease resistance [30,31]. The contents of JA and JA-Ile were substantially higher in *OsSAH*-knockout and lower in *OsSAH*-overexpressing plants than in the ZH17 control plants (Figure 5e), showing synchronous changes with the SA levels. The induction of phytoalexin sakuranetin is considered to be tightly associated with JA accumulation [31]. Sakuranetin was detected at low levels in the knockout plants under normal growth conditions (Figure 5f), consistent with the increase in jasmonate accumulation.

### 2.6. Restricted Induction of OsSAH3 Promoter by M. oryzae

**Figure 6:**
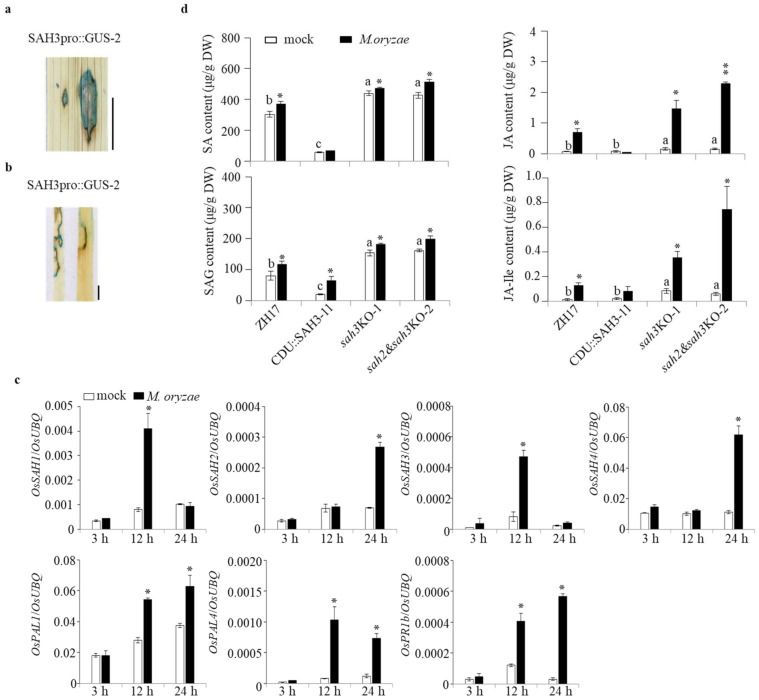
Induction of *OsSAHs* expression and alternation of phytohormone accumulation by *M. oryzae*. (**a**,**b**) Induction of *OsSAH3* promoter expression by *M. oryzae*. The promoter transgenic plants (SAH3pro::GUS-2) were challenged with *M. oryzae* SZ strain by infiltration (**a**) as described in Figure 4 and foliar spray (**b**). Three-week-old rice plants grown in soil were inoculated with *M. oryzae* SZ strain by spraying the spore suspension (5 × 10^5^ conidia/mL containing 0.005% Silwet L-77) or 0.005% Silwet L-77 as the mock treatment. The leaves were sampled for GUS staining, gene expression, and compound determination at the designated time point. Experiments were performed at three biological repeats with similar results. Representative GUS staining images of the lesions stained at 37 °C for 4 h. Bar = 1 cm. (**c**) Induction of *OsSAHs* and defense-related genes by *M. oryzae* SZ. Transcript level was determined by qRT-PCR using *OsUBQ* as the reference gene. (**d**) Compound changes induced by *M. oryzae*. The contents of compounds were determined by LC–MS/MS using D_5_BA as an internal standard. Values are given as means ± SD (n = 3). Asterisks indicate statistically significant differences compared with the mock (Student’s *t*-test, *, *p* < 0.05; **, *p* < 0.01), and columns marked with different letters (a–c) indicate significant differences analyzed by the SPSS software (Duncan’s multiple range test, α = 0.05).

The results showed that SA is required for disease resistance, but SA accumulation is often observed without large changes in pathogen-infected tissues [26]. To determine *OsSAH3* response to *M. oryzae* infection, we inoculated *OsSAH3*-promoter plants (SAH3pro::GUS-2) with *M. oryzae* SZ strain by infiltration. GUS was strongly stained at greenish-infected sites with high GUS density around the lesion borders (Figure 6a). Similarly, GUS staining was mainly around the necrotic lesion borders in SAH3pro::GUS-2 plants 7 d post the spray inoculation (Figure 6b). The results suggest that the expression of SA-responsive genes, such as *OsSAH3*, occurs in a spatiotemporal manner in response to *M. oryzae* infection. Differences in induction of *OsSAH1–4* by *M. oryzae* were observed at the time period tested, along with the expression changes of *OsPAL1* and *OsPAL4* participated in SA biosynthesis, and *OsPR1b* (Figure 6c).

Conversely, the high induction of SA-responsive genes imply an increased accumulation of SA or other active chemicals. We measured compound changes after *M. oryzae* inoculation, and we found that SA and SAG levels were elevated weakly, accompanied by marked increase in jasmonate in *sah3*KO, *sah2*&*sah3*KO, and ZH17 plants 24 h post inoculation (Figure 6d), whereas CDU::SAH3 plants showed negligible changes in SA and JA.

### 2.7. Knockout OsSAH Genes Elevate Basal Resistance

**Figure 7:**
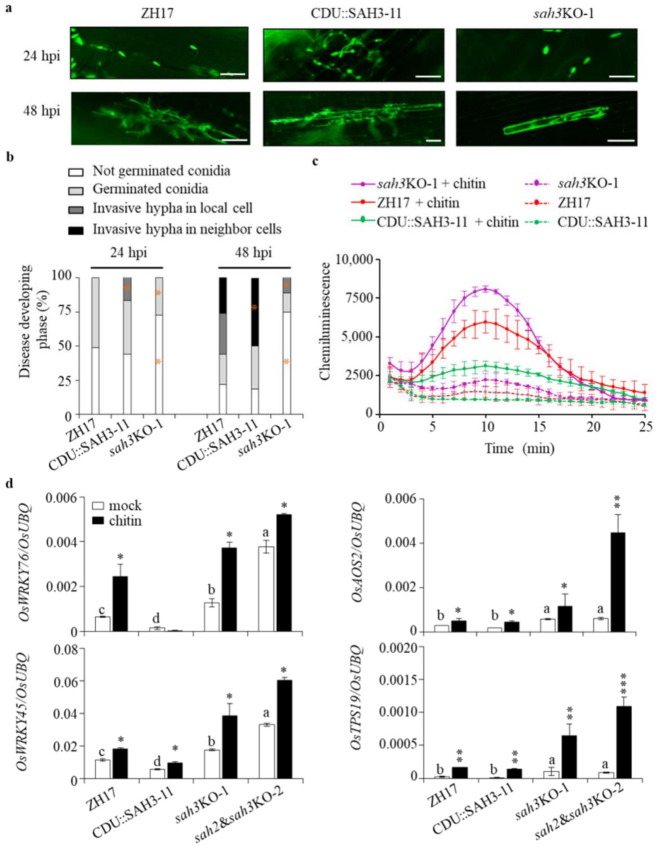
Knockout of *OsSAH3* increased basal immunity. (**a**,**b**) Infection development of *M. oryzae* in the transgenic and control plants. Overexpressing (CDU::SAH3-11) and knockout (*sah3*KO-1) of *OsSAH3* and ZH17 control plants were inoculated with spores of *M. oryzae* SZ-GFP strain (1 × 10^5^ conidia/mL) by infiltration into sheath cells. The spore germination and mycelia growth in the inoculated epidermal layer were evaluated under a fluorescence microscopy. Representative fluorescent images (**a**) and disease development (**b**) were photographed and quantified, respectively, at 24 h and 48 h post the inoculation. More than 100 spores in each line were analyzed. Data from two independent experiments are shown. Asterisks indicate statistically significant differences compared with ZH17 of the same developing stage (Student’s *t*-test, *, *p* < 0.05). Bar = 50 μm. (**c**) chitin-induced ROS burst in the transgenic and control plants. Rice leaf disks were treated with chitin or water. Induction of ROS was detected with a chemiluminescence assay using luminol as the substrate. Values represent means ±SD (*n* = 3). (**d**) Induction of the defense-related genes by chitin. Ten-day-old rice plants, cultured hydroponically in 1/2 MS, were treated with chitin for 1 h and sampled for total RNA isolation. Gene expression was determined by qRT-PCR using *OsUBQ* as the reference gene as described in Figure 3. Results from a representative experiment are shown. Experiments were biologically repeated twice with similar results. Asterisks indicate statistically significant differences compared with the corresponding mock using student’s *t*-test (**, *p* < 0.01; ***, *p* < 0.001). Columns marked with different letters (a–d) indicate significant differences, as analyzed by the SPSS software (Duncan’s multiple range test, α = 0.05).

To obtain more information about SA effects on rice disease resistance, we investigated the early infection process of the GFP-labeled *M. oryzae* SZ strain (SZ-GFP) in the transgenic and control plants. Under visual observation, CDU::SAH3-11 plants presented more germinated spores than *sah3*KO-1 mutant 24 h post infection of the SZ-GFP conidia into the leaf sheaths (Figure 7a,b). Consequently, the germinated conidia invaded more into the neighboring cells in CDU::SAH3-11 than in *sah3*KO-1 plants 48 h after the infection, suggesting that the decrease in SA favors *M. oryzae* spore germination and spread.

SA and JA have priming effects that enhance plant disease resistance [32]. The elevated levels of SA and JA in *OsSAH*-knockout plants prompted us to investigate the defense responses in transgenic plants. The chemiluminescence signal, representing ROS accumulation, induced by chitin was much stronger in the *sah3*KO-1 plants than in the ZH17 control and CDU::SAH3-11 plants, in which CDU::SAH3-11 showed slight ROS induction (Figure 7c).

*OsWRKY45* and *OsWRKY76* are SA-responsive genes. The basal levels of *OsWRKY45* and *OsWRKY76* expression were significantly higher in the knockout mutants and lower in *OsSAH3*-overexpressing plants compared with ZH17 control plants (Figure 7d). After chitin treatment, *OsWRKY45* and *OsWRKY76* transcription levels remained markedly higher in the knockout mutants than in the overexpressing- and control plants (Figure 7d). Similarly, *OsAOS2*, a JA biosynthetic gene, and *OsTPS19*, a JA-responsive gene encoding limonene synthase, were induced more strongly by chitin in the knockout mutants than in ZH17 and CDU::SAH3-11 plants (Figure 7d). In summary, knockout of *OsSAHs* elevated the accumulation of SA and jasmonate and caused a priming effect on gene expression, leading to prevention of pathogen infection at an early stage.

## 3. Discussion

SA is a key signaling molecule involved in plant defense responses against pathogens. In contrast to the induction of SA in *Arabidopsis* and tobacco, the high basal levels of SA in rice cultivars are noteworthy for their role in plant defense and development. SA can be metabolized in both reversible and irreversible ways to maintain SA homeostasis [12,15,33]. In this study, overexpression of *OsSAH2–3* decreased SA content, whereas the knockout of *OsSAHs* increased SA accumulation (Figure 5c), suggesting that a new SA homeostasis with a high SA content is reached in the knockout mutants. Elevated levels of SA are associated with hypersensitive responses in lesion-mimicking mutants [34]. Meanwhile, SA accumulation was dramatically high in some autoimmune mutants with no spontaneous lesion formation, such as *suppressor of npr1-1, constitutive 1* (*snc1*), indicating that high levels of SA alone are not sufficient to activate cell death [35]. Moreover, SA functions as a priming agent to potentiate ROS production and defense-related gene expression in soybean cells [36]. Increased SA and jasmonate levels in *OsSAH*-knockout plants led to increased ROS accumulation and defense-related gene expression induced by chitin, whereas overexpression of *OsSAH3* alleviated defense reactions, including ROS production (Figure 7c). Consequently, we observed inhibition of *M. oryzae* spore germination and invasive hypha spread into neighboring cells in *sah3*KO-1 compared with *OsSAH3*-overexpressing and wild type plants (Figure 7a,b). In rice *aim1* mutant with defective root growth, reduced SA levels are associated with decreased ROS production in the roots [24]. Conversely, *NahG* rice shows reduced endogenous SA but increased levels of ROS and spontaneous lesion formation [26]. Therefore, *NahG* rice plants are hyperresponsive to oxidative damage induced by *M. oryzae* or paraquat treatment. Since phenolic catechol may trigger ROS generation, the susceptibility to oxidative damage of *NahG* rice plants may be due to increased titers of catechol and, hence, ROS intermediates in the plant [37].

Cross-talk between SA and JA signaling pathways has been demonstrated to occur in multiple layers of regulation. Generally, SA signaling plays a crucial role in resistance against biotrophic and hemibiotrophic pathogens, whereas JA signaling contributes to defense against necrotrophic pathogens and herbivorous insects [28]. Studies have shown the antagonistic effects of SA and JA pathways in *Arabidopsis*, as SA application suppresses JA biosynthetic genes [38]. Conversely, the bacterial *Pseudomonas*-secreted phytotoxin coronatine, acting as a JA-Ile mimic, activates three NAC transcription factors, which suppress *ICS1* and increase the SA methyl transferase gene *BSMT1*, leading to reduced SA levels [39]. However, the rice *oshpl3* mutant, which encodes a hydroperoxide lyase, shows overproduction of JA and SA and enhanced resistance to the bacterial blight pathogen *Xoo* [40,41]. Recently, the *RESISTANCE OF RICE DISEASE1* mutant has been shown to contain elevated SA and JA levels, which confers resistance to multiple pathogens in rice [42]. The knockout of *OsSAH* genes elevated the basal levels of SA and jasmonate and conferred resistance against both hemibiotrophic and necrotrophic pathogens (Figure 4 and Figure 5). On the contrast, overexpression of *OsSAH3* compromised the induction of JA and JA-Ile by chitin treatment. The results suggest that a synergistic effect of SA and JA exists under certain conditions in rice [43]. Jasmonate is required for defense against the hemibiotrophic pathogen *M. oryzae* [21], and application of JA after *M. oryzae* infection can alleviate the disease symptoms [44]. It is reasonable to expect that the activated JA signaling pathway in *sah2*–*3*KO plants positively regulates the resistance to necrotrophic pathogens, such as *R. solani* and *B. oryzae* (Figure 4c,d, Figures S8 and S9). Furthermore, DELLA proteins have opposite functions against different lifestyles of invading pathogens in *Arabidopsis* and rice. In *Arabidopsis*, DELLA modulates immunity by promoting JA signaling and antagonizing SA, thus increasing resistance to the necrotrophic fungus *Alternaria brassicicola* and susceptibility to the hemibiotrophic bacterium *Pseudomonas syringae* pv. *tomato* [45]. In rice, DELLA Slender Rice1 (SLR1) acts as a positive regulator of hemibiotrophic resistance by integrating and amplifying SA- and JA-mediated defense signaling [46]. Collectively, synergistic SA–JA interactions in rice may provide a common defense pathway for broad-spectrum disease resistance.

The synergistic interaction between SA and JA has also been shown during ETI in *Arabidopsis* as a unique interplay [25,47]. JA accumulates at high levels when ETI is evoked and contributes positively to ETI. Induction of JA biosynthesis following SA accumulation is activated through the SA receptors NPR3 and NPR4, which mediate the effect by promoting degradation of the JA transcriptional repressor JAZs [25]. Alternatively, Betsuyaku et al. [48], using a time-lapse imaging assay of defense-related gene promoters, showed that the JA signaling pathway is activated in the vicinity of the central SA-active cells during ETI, indicating the spatiotemporal dynamics of the SA–JA relationship. Restricted inductions of *OsSAH3*-promoter activities by *M. oryzae* infection suggests that SA and JA levels may be elevated around the infection foci in a pattern similar to that of ETI in *Arabidopsis* (Figure 4) [48]. The localized activation of the promoter activities might provide an explanation for the observation of weak or no change in SA levels in rice shoots following pathogen infection (Figure 4) [26]. The increased level of SA in the infection sites is probably buffered by the large basal SA pool.

There were three and four SAHs in *Arabidopsis* and rice genomes, respectively (Figure S3). The *DMR6* mutant was isolated as a mutant with enhanced resistance to downy mildew, and the *DLO1*/*S3H1* mutant is associated with senescence [9,11]. *DMR6* and *DLO1*/*S3H1* show differences in their spatial expression patterns in *Arabidopsis* leaves infected with *H. parasitica*, whereas *DLO2* is not expressed in leaves [10]. SA is over accumulated in the *dmr6 dlo1*/*s3h1* double mutant compared with the *dmr6* mutant, displaying immunity to pathogens, growth retardation, and early senescence phenotype [10,12]. Similarly, the rice *SAH* single and double mutants showed elevated SA and jasmonate levels and attenuated disease susceptibility to different lifestyle pathogens (Figure 2 and Figure 3). The results demonstrated that each *SAH* in *Arabidopsis* and rice has distinct activities based on their spatiotemporal expression patterns, even though partial redundancy may exist [10].

SAHs of *Arabidopsis* and rice are divided into two subclades (Figure S3), suggesting that these genes exist before species divergence and evolve separately in their genomes. OsSAH3 with only SA5H activity groups with DLO1/S3H1 and DLO2. However, MDR6 (with high SA5H activity) is grouped with the other three OsSAHs, as it has relatively much lower SA5H activity compared with that of OsSAH3 (Table 1). *OsSAH2* and *OsSAH3,* as well as *DLO1*/*S3H* and *DLO2,* are localized in the vicinity of their chromosomes and were potentially gained by duplication. Functional divergence of OsSAH2 and OsSAH3 was observed at the biochemical level (Table 1; Figure 2). OsSAH3^P292A^ mutant decreased 2,5-DHBA production; however, the Ala to Pro substitution at the P sites of OsSAH2 and DMR6 completely blocked 2,5-DHBA activity and suppressed 2,3-DHBA production (Figure 2b,c). The data suggest that the relatively fixed side chain of Pro compared with that of Ala had more severe effects on subclade-I than in subclade-II proteins. Site P is between the RxS motif and conserved Phe amino acid, which are in contact with 2OG and possibly associated with the co-substrate SA, respectively [18]. The change in amino acid volume at site P might affect substrate docking and/or oxygen transfer vs. Fe ion. The results revealed that site P plays a critical role in enzymatic activity and specificity. As SA forms an intramolecular hydrogen bond between the carboxyl and the ortho hydroxyl groups [49], SA conformation probably hinders the rotation of the benzene ring within the OsSAH active pockets. Therefore, the ratio of 3- to 5-hydroxyl products was determined when the substrate was initially associated with the OsSAH enzymes. Furthermore, we noted that OsSAH3 has no activity for 2-Cl-BA, but low activity for 3-Cl-SA, implying that the ortho hydroxyl group of SA may be specifically required for binding (Figures S2 and S4c). There are reports documenting that a single amino acid substitution affects substrate docking and product specificity. Modification of the JA receptor with Ala to Val substitution in the JA-Ile-binding pocket of CORONATINE INSENSITIVE1 (COI1) protein greatly reduces sensitivity to the JA-mimicking toxin, coronatine, but it remains sufficient for endogenous JA signaling [50]. OsTPS19 and OsTPS20 are two limonene synthases, in which the functional variation is determined by a single amino acid (Ala vs. Ser) localized in the active site cavity [27]. Nevertheless, the generation of SAH crystal structures may provide clear images of the SAH active cavity. 

## 4. Materials and Methods

### 4.1. Generation of Transgenic Plants

The coding sequences of *OsSAH2*-*3* were amplified from ZH17 (*Oryza sativa* L. Zhonghua 17) cDNA using gene-specific primers (Table S1), fused with 3 × Myc tag at the 3’ end, and placed under maize *ubiquitin* promoter. For gene knockout, the gene-specific target sequence of each gene was selected and under the control of the U3 promoter in the pOsCas9 vector. For the *OsSAH3* (SAH3pro::GUS) promoter construct, the sequence of approximate 2.0 kb, including the translation start site, was inserted into a modified pCambia1301 (Cp-GUS) vector [25]. 

The verified plasmids were transformed into *Agrobacterium tumefaciens* EHA105 for rice transformation. The transgenic plants were generated from the immature seeds of ZH17 by the *Agrobacterium*-mediated transformation method [25]. Transgenic plants were detected by PCR amplification and verified by sequencing.

### 4.2. Plant Growth and Treatments

The seeds of transgenic and control plants were surface sterilized and germinated in ½ Murashige and Skoog (MS) medium. The seedlings were cultured in ½ MS liquid medium at 28 °C with a 12-h-light photoperiod and treated with 5 mM MES (4-morpholine ethanesulfonic acid, pH 5.8) buffer containing 500 μM SA, 100 μM MeJA, 500 μM M BTH, or 200 μg/mL chitin, and the same volume of DMSO solvent was used as the control.

### 4.3. Pathogen Inoculation

Three-week-old rice plants were inoculated with a virulent *M. oryzae* SZ strain by spraying the spore suspension (5 × 10^5^ conidia/mL containing 0.005% Silwet L-77) as described by Liu et al. [25]. For injection, rice plants at the tillering stage (about three-months old) were injected with the spore suspension (1 × 10^5^ conidia/mL) to the center of each leaf sheath, and the newly grown leaves were collected for disease severity evaluation. To observe infection process, the spores of EGFP-tagged *M. oryzae* SZ-GFP strain (1 × 10^5^ conidia/mL) were infiltrated into the leaf sheath, as described previously [51]. The inoculated sheaths were kept in a petri dish containing wet filter paper. The inner layer of the leaf sheaths were examined with a fluorescence microscope (Nikon Ti-E, Nikon Co., Ltd., Tokyo, Japan) at 24- and 48 h post the inoculation. 

To evaluate resistance against bacterial blight pathogen, three-month-old rice plants were inoculated with *Xoo* J18 (OD_600_ = 0.8) by the leaf-clipping method [25]. Disease severity was estimated by measuring lesion length 18 d after the challenge.

Three-week-old rice plants were inoculated with *B. oryzae* LW by foliar spraying (5 × 10^5^ conidia/mL spores containing 0.005% Silwet L-77), in which the *B. oryzae* LW strain was isolated from the natural infected rice leaves. Disease severity was evaluated by PCR amplification of the relative biomass. Quantification was performed with primers from *B. oryzae* 26S rDNA and rice *ubiquitin* gene (*OsUBQ*). Template DNAs from the inoculated leaves were collected 7 d after the inoculation. Number of disease spot infected naturally by *B. oryzae* in paddy field was counted as described previously by Zanão Júnior et al. [52].

To evaluate resistance against rice sheath blight, five-week-old rice plants were inoculated with *R. solani* XN and a weak virulent uninucleate *Rhizoctonia* JN isolate grown on PDA with slices of filter papers (about 10 mm × 4 mm) on the agar for five days. The filter papers, grown over by XN or JN, were pinned around the sheath bottoms of rice. Lengths of disease lesion were measured after four days inoculation. 

### 4.4. ROS Detection

ROS detection was followed as described previously [53]. Six-week-old rice leaves were cut into 4-mm-diameter disks and equilibrated in distilled water for about 10 h. The assay mixtures contained 100 μL of luminol (Immun-Star horseradish peroxidase substrate, Bio-Rad, Hercules, CA, USA), 1 μL of horseradish peroxidase and 8 nM chitin (hexa-N-acetylchitohexaose) or water. The reactions were started by adding three leaf disks into the mixtures in a 1.5 mL microcentrifuge tube (Axygen Scientific, Union City, CA, USA). Luminescence was detected with a luminometer (GloMax 20/20, Promega, Madison, WI, USA) for 25 min.

### 4.5. Protein Expression and Enzyme Assays

*DMR6* sequence was amplified from cDNAs of *A. thaliana* leaves. Mutations were generated by PCR-based site-directed mutagenesis. Each *OsSAH1*–*4*, *DMR6*, and their mutants, was cloned into a modified pGEX-tag vector with 3 × Myc at the C-terminus of the recombinant protein [25]. After transformation of each plasmid into *Escherichia coli* BL21 (DE3), the protein expression was induced by addition of 0.2 mM IPTG grown at 28 °C for 6 h. The recombinant proteins were purified using Glutathione Sepharose 4B (GE Healthcare, Chicago, IL, USA). 

The enzymatic activity assay was performed as described previously [11]. Briefly, a total volume of 100 μL reaction mixture contains 5 μg recombinant protein in the reaction buffer (1 mM 2-oxoglutaric acid, 1 mM sodium ascorbate, 0.4 mM FeSO_4_, 0.1 mg/mL catalase, 5 mM DTT, 50 mM phosphate buffer at pH 6.8) with different concentrations of SA or other compounds tested and reacted at 40 °C for 30 min. The reaction was stopped by adding two volumes of acetonitrile and boiled for 1 min. Then, the mixture was extracted with 200 μL ethyl acetate for twice, the combined organic solutes were dried by nitrogen gas and dissolved in 50 μL of 90% aqueous methanol containing 0.1% formic acid for product detections. For optimal assay conditions, we performed the assays in citrate buffer (pH 5.8), phosphate buffer (pH 6.3, 6.8, and 7.3), or Tris-HCl buffer (pH 7.8) at 40 °C and in phosphate buffer (pH 6.8) at different temperatures. Substrate inhibition kinetics were obtained as described by Kutsuno et al. [54].

### 4.6. Determination of Metabolites

Compound isolation and determination were described previously [55]. Briefly, the freeze-dried plant tissues (approximate 25 mg for each replicate) were extracted with 1 mL 90% aqueous methanol containing 0.1% formic acid and an internal standard of 20 ng D_5_BA. After dry of the supernatants by nitrogen gas, the residues were dissolved in 0.1 mL of 90% aqueous methanol containing 0.1% formic acid for LC–MS/MS. For targeted MS/MS analysis, collision voltage was applied with 20 V for reliable fragmentations. Chemicals were separated by a C_18_ column (2.1 mm × 150 mm, 3 μm, Phenomenex) on Agilent 1260 separation module (Agilent Co. Ltd., Santa Clara CA, USA). The elution conditions were at a flow rate of 0.25 mL/min with a gradient program of 5% acetonitrile for 2 min, up to 25% in 8 min, then to 70% in 30 min, and then the column was washed and equilibrated to the initial conditions. 

### 4.7. Structural Modeling and Substrate Docking

Conformations of OsSAH2, OsSAH3, DMR6, and OsSAH3 variants were simulated based on the F6’H1 (PDB ID: 4XAE) crystal structure using SWISS-MODEL [56]. The highest score of each protein from global model quality estimation was selected for docking with salicylic acid (PubChem CID: 338) and 2-oxoglutaric acid (PubChem CID: 51). Substrate docking was performed in the conserved active sites of the protein via AutoDock Vina [57]. Interactions between protein and substrate were analyzed via Protein-Ligand Interaction Profiler [58] and visualized in PyMOL.

### 4.8. GUS Staining

GUS (beta-glucuronidase) staining was performed in the staining buffer (50 mM sodium phosphate at pH 7.0, 0.5 mM K_3_Fe[CN]_6_, 0.5 mM K_4_Fe[CN]_6_, 10 mM EDTA-2Na, 0.1% Triton X-100, 0.5 mg/mL X-gluc) for a designated time.

### 4.9. Reverse Transcribed Quantitative (qRT-PCR) Analysis

After removing possible DNA contaminations, two micrograms of total RNA were reverse transcribed with random hexamers and oligo(dT)_18_ primers using M-MLV reverse transcriptase (Takara, Kusatsu-shi, Japan). The relative transcript levels were quantified using SYBR Green PCR Master Mix (Takara) and normalized to *OsUBQ*. The relative expression level of each gene was analyzed using the delta-delta *C*t method. Gene-specific primers used in qRT-PCR are listed in Supplementary Table S1.

## 5. Conclusions

We showed the expression patterns of the four *OsSAH* genes and revealed three amino acids affecting the enzyme hydroxylation activity and product specificity. Knockout of *OsSAH* genes increased SA homeostasis at an elevated level, and jasmonate content, conferring broad-spectrum disease resistance against both hemibiotrophic and necrotrophic pathogens. Our findings indicate that SA plays a crucial role in the rice immune signaling pathway.

## Figures and Tables

**Table 1:** Kinetic parameters of enzyme reaction.

Products	Protein	Specific Activity *	*K_m_* (μM)	*V_max_* *	*K_si_* (μM)	*K_cat_* (min^−1^)	*K_cat_/K_m_* (s^−1^·M^−1^)	R^2^
2,3-DHBA	SAH1	0.02	41.07 ± 39.93	0.07 ± 0.06	16.49 ± 16.49	0.05 ± 0.04	20.21	0.85
	SAH2	0.03	5.87 ± 3.97	0.05 ± 0.02	14.52 ± 10.36	0.03 ± 0.01	85.18	0.80
	SAH3	nd						
	SAH4	0.02	20.44 ± 13.88	0.05 ± 0.02	24.26 ± 16.79	0.03 ± 0.01	24.51	0.85
2,5-DHBA	SAH1	1.57	57.41 ± 44.01	6.42 ± 3.94	27.13 ± 21.11	4.40 ± 0.65	1277.30	0.91
	SAH2	0.07	12.48 ± 10.96	0.42 ± 0.31	2.14 ± 1.86	0.29 ± 0.21	387.26	0.95
	SAH3	26.37	197.08 ± 19.42	22.28 ± 2.89	365.12 ± 30.58	15.36 ± 1.99	1287.71	0.96
	SAH4	0.21	18.32 ± 6.97	0.52 ± 0.14	26.52 ± 10.71	1.52 ± 0.41	363.90	0.98

Kinetic parameters were obtained from the reactions at pH 6.8 and 40 °C for 30 min. nd, not detectable. * nmol/mg protein/min. Values are given as means ± SD. nd for not detection. * nmol/mg protein/min. *K_m_* for Michaelis constant, *V_max_* for maximum reaction rates, *K_si_* for inhibition constant, *K_cat_* for catalytic rate constant, and *K_cat_*/*K_m_* for catalytic efficiency.

## Data Availability

The data presented in this study are available upon request from the corresponding author.

## Supplementary Materials

The following supporting information can be downloaded at: https://www.mdpi.com/article/10.3390/ijms23031354/s1.
