# Supplementary material for: Salicylic Acid Is Required for Broad-Spectrum Disease Resistance in Rice
Source: Int J Mol Sci. 2022 Jan 25;23(3):1354. doi: 10.3390/ijms23031354 (PMC8836234; doi:10.3390/ijms23031354)
Supplement: Supplementary file 1 [file ijms-23-01354-s001.zip › Supplementary figures.pdf]

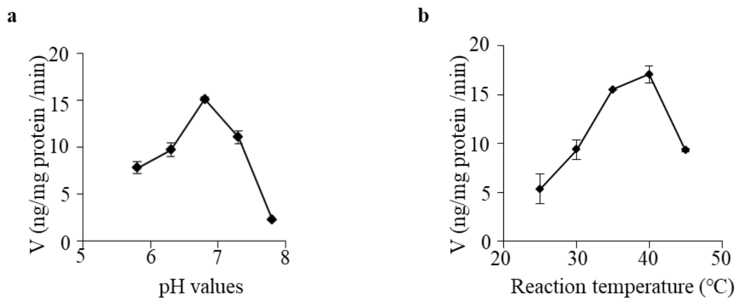

Figure S1. Optimal pH (a) and temperature (b) for OsSAH3 protein. The reactions were conducted in the mixtures of different pH values at 40°C (a) or at different temperatures in the mixture of pH 6.8 (b) using SA as the substrate. The data are presented as means ± SD (n = 3).

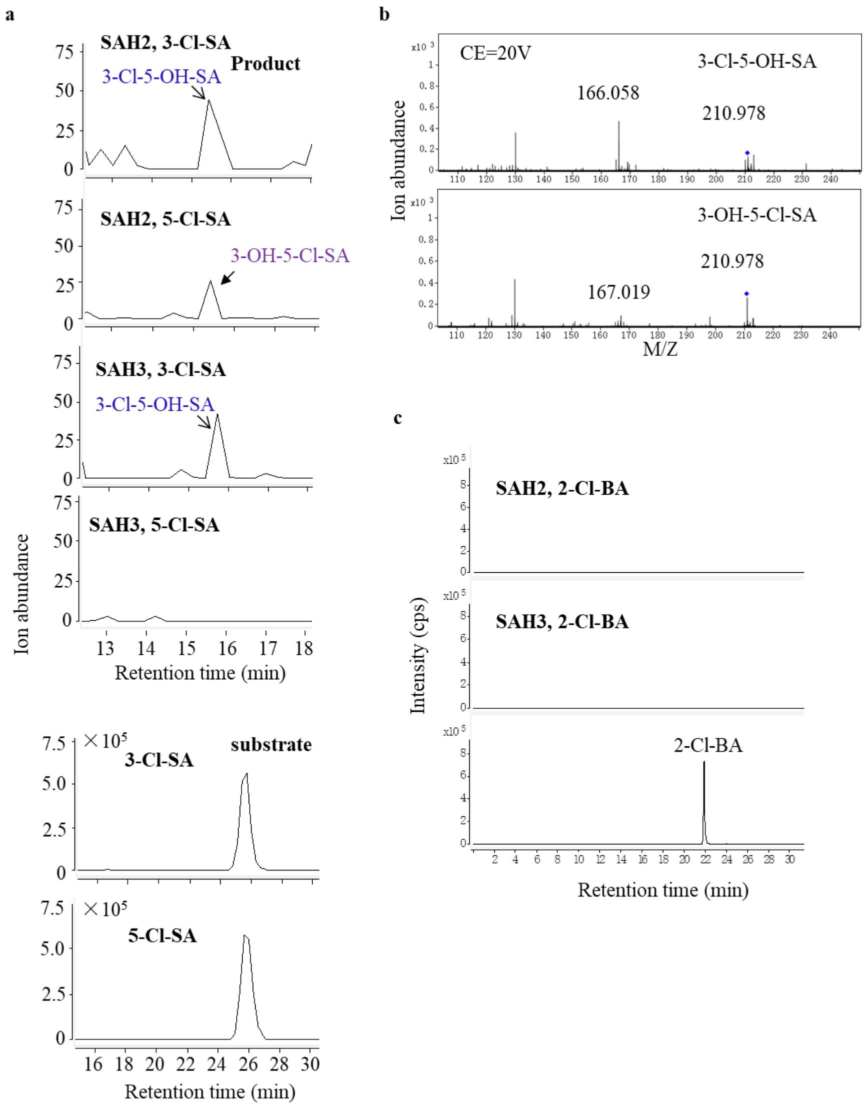

Figure S2. Enzyme activity of OsSAH2 and OsSAH3 toward SA related compounds. (a) Recombinant OsSAH2 and OsSAH3 catalyze 3-Cl-SA and 5-Cl-SA to form hydroxylation product. The reaction mixture was analyzed by LC-MS/MS. Specific ions for 3-Cl-5-OH-SA [M+Na-H]$^-$, 210.978, 166.058 and 3-OH-5-Cl-SA [M+Na-H]$^-$, 210.978, 167.019. (b) Recombinant OsSAH2 and OsSAH3 catalyze 2-Cl-BA, producing no detectable product. CE, Collision energy.

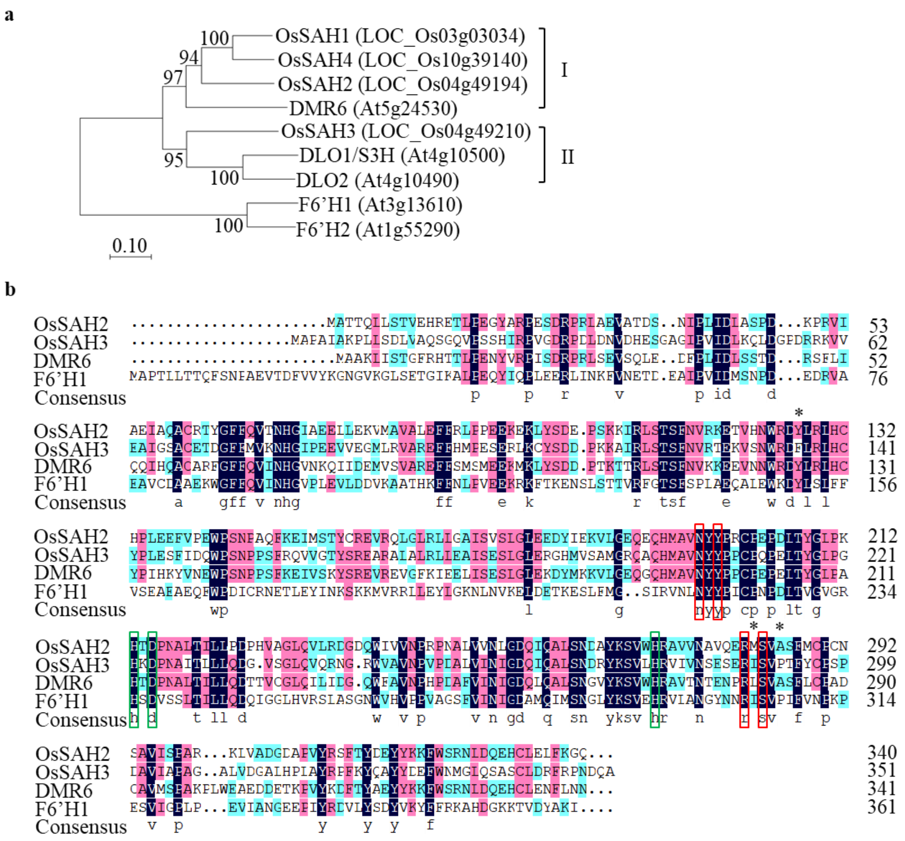

Figure S3. Phylogenetic tree and sequence alignment. (a) Phylogenetic relationship of OsSAH1–4 and homologous proteins in Arabidopsis. Phylogenetic tree was constructed via MEGA7.0 using the neighbor-joining method. Bootstrap values (1000 replicates) are calculated. OsSAH1, OsSAH2, OsSAH4 and DMR6 are clustered in subclade I, while OsSAH3, DLO1 and DLO2 are clustered in subclade II. (b) Sequence alignment of OsSAH2, OsSAH3, DMR6 and F6'H1. Identical residues shaded in dark blue, identity more than 75% in pink, and identity within 50% –75% in cyan. Iron and 2OG binding residues are boxed in green and red, respectively. The mutation sites are marked by stars.

a

OsSAH3 docking with SA and 2OG

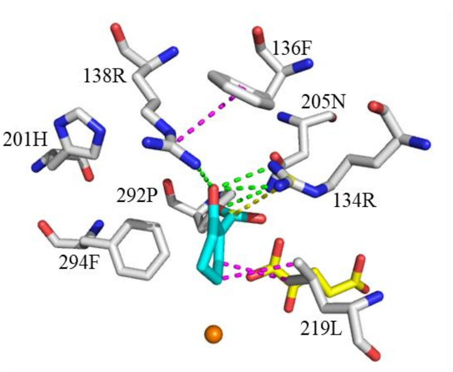

Docking energies (△G): -5.8 kcal/mol
Fe–C5 distance: 4.6 Å

c

OsSAH3 docking with 2-Cl-BA and 2OG

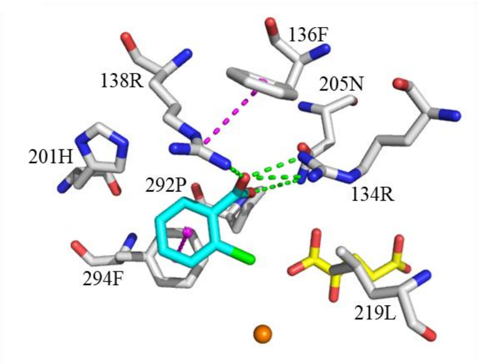

Docking energies (△G): -5.7 kcal/mol
Fe–C3 distance: 5.6 Å

b

OsSAH3$^{F136Y/P292A}$ docking with SA and 2OG

For 2,5-DHBA production

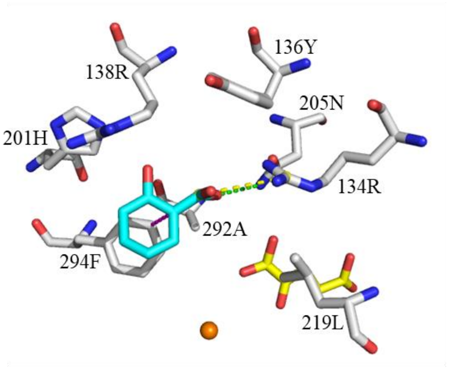

Docking energies (△G): -5.2 kcal/mol
Fe–C5 distance: 5.8 Å

For 2,3-DHBA production

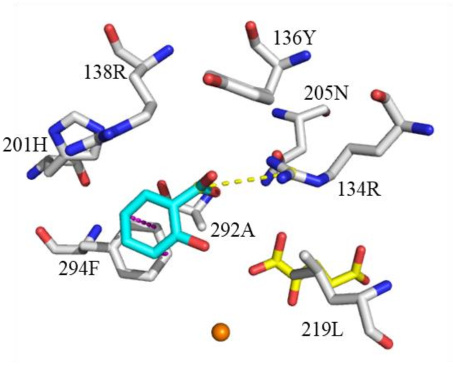

Docking energies (△G): -5.0 kcal/mol
Fe–C3 distance: 5.7 Å

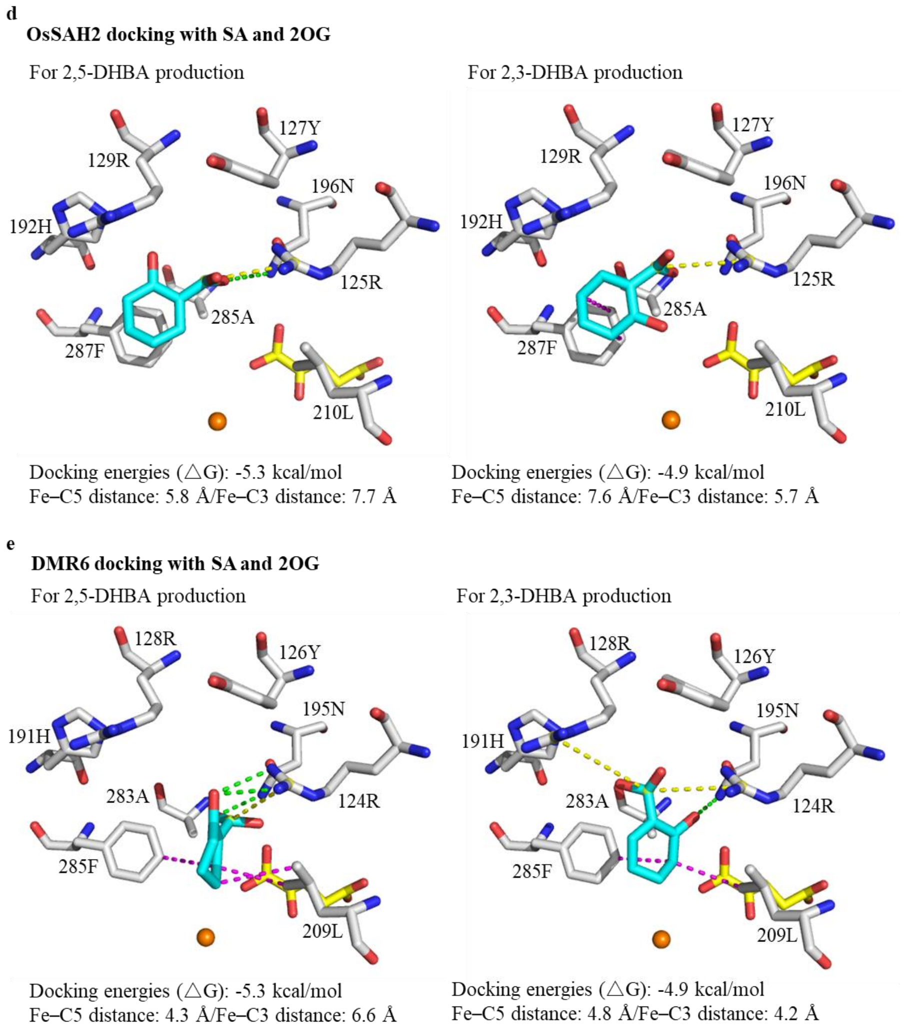

Figure S4. Conformations of SAH docking with substrates. SA and co-substrate 2OG binding to OsSAH3 (a), OsSAH3$^{F136Y/P292A}$ (b), OsSAH2 (d), and DMR6 (e) and (c) OsSAH3 interacting with 2-Cl-BA and 2OG. Conformations were simulated based on the F6'H1 (PDB ID: 4XAE) crystal structure using SWISS-MODEL. Substrate docking was performed by using AutoDock Vina. Interactions between protein and substrate were analyzed via Protein-Ligand Interaction Profiler and visualized in PyMOL. The docking energies and the distance between iron (Fe^{2+}) and the potential hydroxylation position in the benzene ring were shown below the pictures. Amino acids and compounds are presented with the stick model. Number for the amino acid position; amino acid in gray, 2OG in yellow, Fe^{2+} in orange, and SA in cyan; hydrogen bond in green, hydrophobic interaction in magenta, and salt bridge in yellow dash.

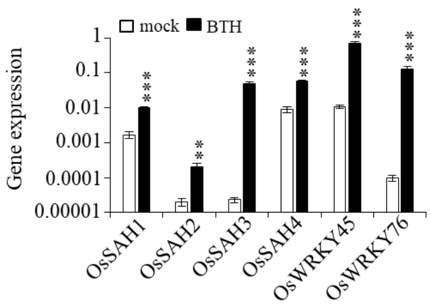

Figure S5. Induction of *OsSAH* genes by BTH. Ten-day-old seedlings cultured hydroponically were treated with 500 μM BTH in 5 mM MES buffer for 6 h, and the mock treatment was received the buffer containing the same volume of DMSO. Gene expression was determined by qRT-PCR using *OsUBQ* as the reference gene. *OsWRKY45* and *OsWRKY76* were used as SA-responsive genes. Values are means ± SD (n = 3). Asterisks indicate statistically significant differences compared with the mock using student's *t*–test (**, P < 0.01; ***, P < 0.001).

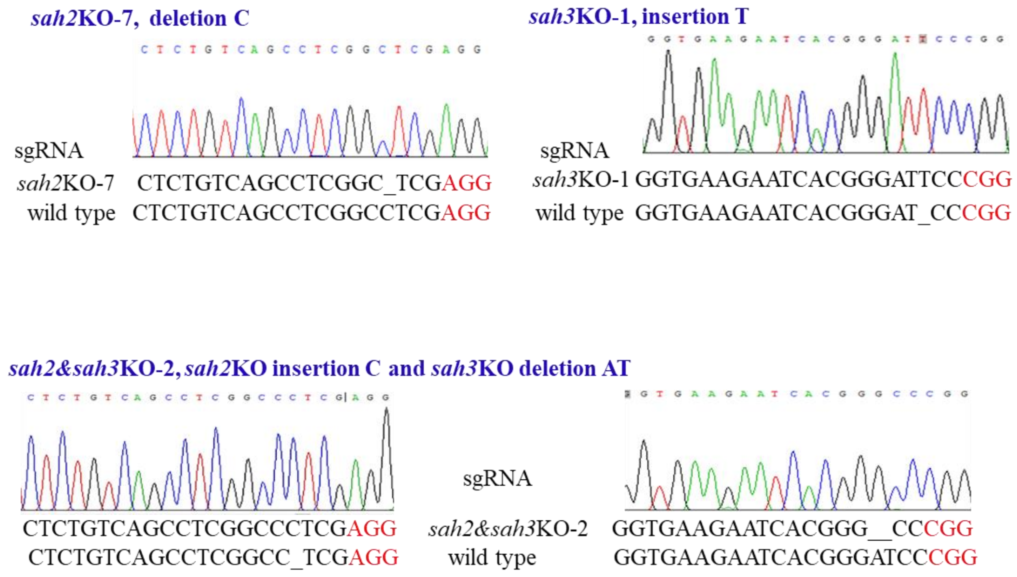

Figure S6. Information of knockout *OsSAH* genes. The sequencing profile of the mutant is shown. "_" for missing nucleotide(s) compared between the sgRNA (wild type) and the mutant

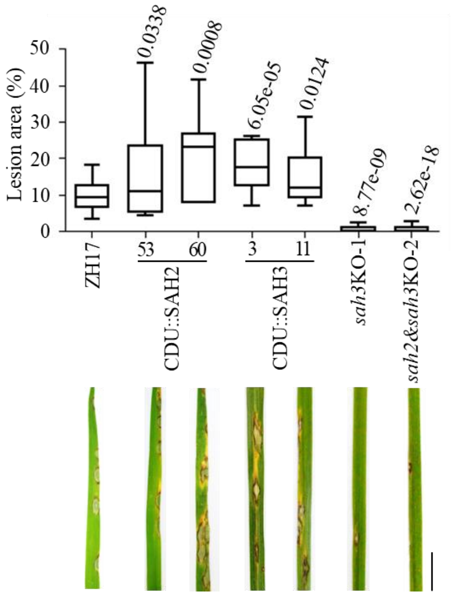

Figure S7. Effect of *OsSAH* genes on resistance against *M. oryzae*. The overexpressing (CDU::), knockout (KO) and control (ZH17) plants were inoculated with the spores of *M. oryzae* SZ strain by foliar spraying ($5{\times}10^5$ conidia/mL). Evaluation of disease severity and photography taken were conducted at 7 d post the inoculation. The median was the cross line in each boxplot showing the lesion area. Results from two biological experiments are shown. P value evaluated using the student's *t*–test is above the boxplot. Bar= 1cm.

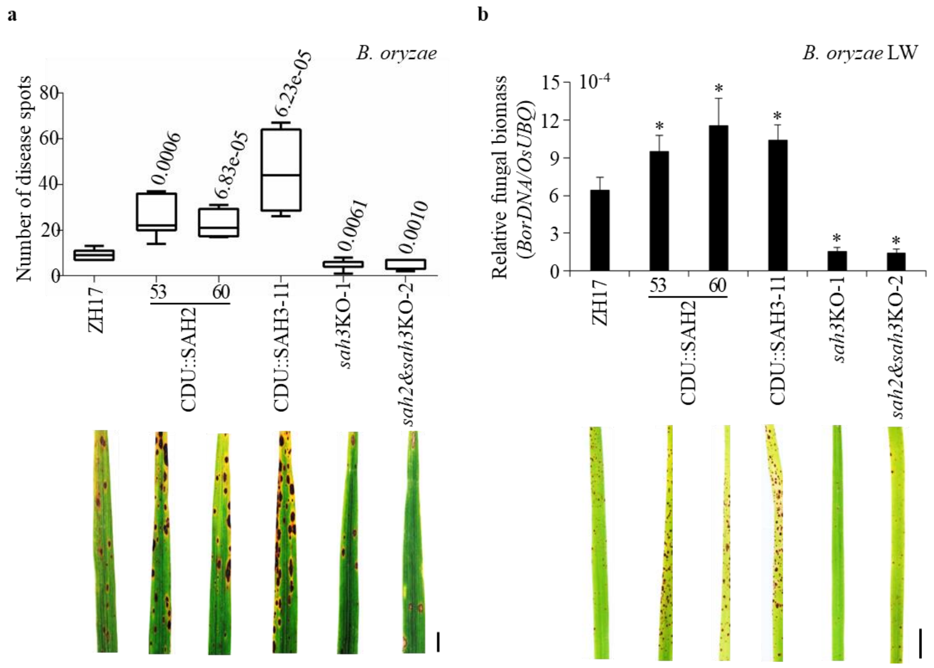

Figure S8. Effect of *OsSAH* genes on resistance against *B. oryzae*. Disease symptom and severity infected naturally in paddy field (a) and by foliar spraying of spores from *B. oryzae* LW (b). (a) The transgenic and ZH17 control plants were grown in paddy field and infected naturally. Evaluation of disease severity and photography taken were conducted about five months (i.e. just before seed harvest) after the seed germination in year 2018. Lesion numbers of *B. oryzae* were determined on 20 cm of each leaf started 2 cm from the tip (n = 10). Then, the *B. oryzae* was isolated and named as LW isolate. P value evaluated using the student's t-test is above the boxplot. (b) Three-week-old plants were inoculated with *B. oryzae* LW isolate (5×10^5 conidia/mL) by foliar spraying; Evaluation of disease severity and photography taken were conducted at 7 d post the inoculation. The relative fungal biomass was evaluated by qPCR using *B. oryzae* 26S rDNA and rice Os*UBQ* gene. Results from a representative experiment are shown. Experiments were biologically repeated three times with similar results. Asterisks indicate statistically significant differences compared with ZH17 using student's *t*–test.

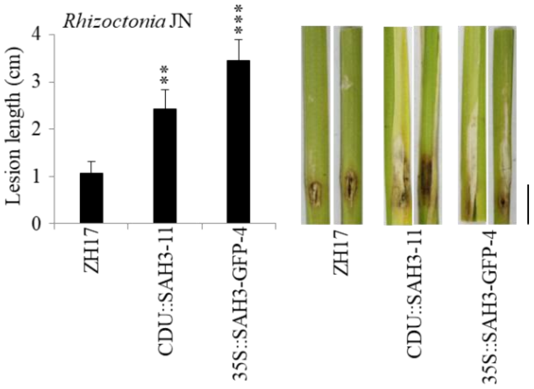

Figure S9. Effect of *OsSAH3* on resistance against *Rhizoctonia* JN isolate. Disease phenotypes and lesion lengths of *Rhizoctonia* JN. Five-week-old rice plants were inoculated with necrotrophic fungus JN isolate. A slice of filter paper containing the *Rhizoctonia* mycelia was pinned around the sheath bottom. Lesion lengths were measured 4 d after the inoculation. Results from two biological experiments are shown. P value evaluated using the student's t-test is above the boxplot. Values are means ± SD (n = 5). Significance was evaluated using the student's *t*–test (*, $P < 0.05$; **, $P < 0.01$; ***, $P < 0.001$). Prefix CDU for overexpressing gene; suffix KO for knockout gene; and ZH17 for wild type plant. Bar = 1 cm.
